# Impact of phenolic compounds on progression of *Xylella fastidiosa* infections in susceptible and *PdR1*-locus containing resistant grapevines

**DOI:** 10.1371/journal.pone.0237545

**Published:** 2020-08-07

**Authors:** Christopher M. Wallis, Adam R. Zeilinger, Anne Sicard, Dylan J. Beal, M. Andrew Walker, Rodrigo P. P. Almeida

**Affiliations:** 1 Crop Diseases, Pests and Genetics Research Unit, USDA-ARS San Joaquin Valley Agricultural Sciences Center, Parlier, California, United States of America; 2 Department of Environmental Science, Policy, and Management, University of California, Berkeley, California, United States of America; 3 Department of Viticulture and Enology, University of California, Davis, California, United States of America; University of Idaho, UNITED STATES

## Abstract

Pierce’s disease is of major concern for grapevine (*Vitis vinifera*) production wherever the bacterial pathogen *Xylella fastidiosa* and its vectors are present. Long-term management includes the deployment of resistant grapevines such as those containing the *PdR1* locus from the wild grapevine species *Vitis arizonica*, which do not develop Pierce’s disease symptoms upon infection. However, little is understood about how the *PdR1* locus functions to prevent disease symptom development. Therefore, we assessed the concentrations of plant defense-associated compounds called phenolics in healthy and *X*. *fastidiosa*-infected *PdR1*-resistant and susceptible grapevine siblings over time. Soluble foliar phenolic levels, especially flavonoids, in *X*. *fastidiosa*-infected *PdR1*-resistant grapevines were discovered to be significantly lower than those in infected susceptible grapevines. Therefore, it was hypothesized that *PdR1*-resistant grapevines, by possessing lowered flavonoid levels, affects biofilm formation and causes reduced *X*. *fastidiosa* intra-plant colonization, thus limiting the ability to increase pathogen populations and cause Pierce’s disease. These results therefore reveal that differences in plant metabolite levels might be a component of the mechanisms that *PdR1* utilizes to prevent Pierce’s disease.

## Introduction

Pierce’s disease, caused by the bacterium *Xylella fastidiosa* ssp. *fastidiosa*, is a major threat to susceptible grapevines in warmer climates worldwide where both the pathogen and its xylem sap-feeding vectors are present [1–3]. Losses in one region alone, California, can reach $104 million per year [4]. Current management involves the use of insecticides to reduce vector populations, but development of pesticide resistance compromises this control method [5]. Likewise, insecticides to manage pathogen vectors, especially over long periods of time, potentially harm the environment, agroecosystem, and biodiversity [6]. Long-term, durable control of Pierce’s disease ultimately lies in development of resistant hosts, in which bacterial pathogen populations are reduced, or tolerant hosts, which prevent significant losses due to disease [7–8]. These resistant or tolerant hosts could be successfully deployed in areas where Pierce’s disease is a problem to mitigate the otherwise large losses associated with the disease.

A variety of approaches have been made to create resistant or tolerant grapevines to Pierce’s disease. A combination of both traditional breeding and transgenics have resulted in different cultivars that have exhibited often durable resistance to *X*. *fastidiosa* infection [9–10]. Transgenic grapevines hold promise as an option for resistant cultivars as they were created with specific, researched methods in mind to directly impact the progression of *X*. *fastidiosa* infections [11–13]. Although transgenics can be utilized as rootstocks so the fruit-producing scion remains wild type [10], there are hurdles to widespread use including public apprehension, marketing challenges, and grower acceptance.

Traditional breeding efforts also have yielded resistant and tolerant grapevines without the market acceptance hurdles of transgenics. However, little is known about the mechanisms of resistance. Resistance to Pierce’s disease is present in a wide variety of *Vitis* spp., with the commercialized European grapevines (*Vitis vinifera*) considered mostly susceptible to *X*. *fastidiosa* ssp. *fastidiosa*. Among grapevines that do not develop Pierce’s disease when infected with *X*. *fastidiosa* ssp. *fastidiosa* is the western North American species *V*. *arizonica* [14]. A genetic locus was found that is associated with the source of defense against infection called *PdR1* (Pierce’s Disease Resistance 1), which confers good resistance (lowered bacterial populations and reduced to no symptoms) in hybrids with this locus that are infected with *X*. *fastidiosa* [9, 14]. This locus has been utilized in screening programs with multiple backcrosses with *V*. *vinifera* grapevines to create a viable commercial grape [9], which is now at the 97–98% *V*. *vinifera* genetic purity level.

Despite the promise of *PdR1* for management of Pierce’s disease more information about its mechanism(s) is desired to evaluate and ensure its long-term viability. Research was thus conducted to explore the host responses to *X*. *fastidiosa* ssp. *fastidiosa* of *PdR1*-containing and *PdR1*-missing sibling grapevine segregates. Plants possess a wide variety of compounds to combat infections [15]. Among these compounds is a class termed phenolics, which have diverse functions ranging from roles in plant growth and development (such as cell wall thickening, hormone production, pigmentation), reproduction (pigmentation, fruit flavoring, fruit protection), and defense against stressors (osmoregulation, UV protection, anti-herbivory roles, and antimicrobial activity) [15].

Resistance to *X*. *fastidiosa* in transgenics may be achieved by disrupting the bacteria’s ability to affect host physiology, in particular cell walls and induced host responses of which both are related to phenolic compounds [10]. Furthermore, previous work demonstrated that phenolics levels observed in grapevines were related to Pierce’s disease progression, and differences among susceptible cultivars in terms of severity of symptoms over time were linked to phenolics [16–17]. In these cases, lowered levels in certain grapevine cultivars of specific phenolic compounds (mostly catechins/procyanidins) appeared to result in lowered symptom development over 6 months [16–17]. A potential role for phenolics was observed when a lipopolysaccharide O-antigen was removed from wild type *X*. *fastidiosa* resulting in a mutant (*wzy*) strain that better triggered host innate immune responses geared towards biological stresses [18], which in turn upregulated genes related to many different types of phenolics. In that experiment the wild type bacteria triggered abiotic-associated responses that appear critical for Pierce’s disease development from *X*. *fastidiosa* infections [18]. Certain phenolics play important roles in either of these stress responses with cell-wall related phenolics such as hydroxycinnamic acids likely related to abiotic responses such as tylose formation deemed important in Pierce’s disease development [19–21]. In contrast, stilbenoids are related to biological stress-induced, pathogen-specific responses to limit pathogen growth [15]. Specific phenolics also can play a role in both abiotic and biotic stress responses such as flavonoids [22], and individual phenolics are not mutually exclusive to abiotic or biotic stress responses.

We hypothesized a role for phenolic compounds in imparting *PdR1* locus-containing grapevine selections resistance to infection by *X*. *fastidiosa* and the related observations of partial resistance. Compound levels also were hypothesized to be greater in grapevines that were infected with the bacterial pathogen as grapevine hosts mount a defense or deal with the consequences of *X*. *fastidiosa* infections, with levels increasing over time as well. Ultimately, findings in this study would confirm which phenolic compounds play a role in how the *PdR1* locus functions and provide insights into innate grapevine responses to *X*. *fastidiosa* infection. Such information could be used to screen other grapevine selections with wild *Vitis* spp. backgrounds for similar traits, which would suggest such selections also have resistance to *Xylella* infections.

## Material and methods

### Plant materials and growth conditions

Greenhouse experiments were conducted at the University of California, Berkeley, Oxford Tract greenhouses. Pierce’s disease resistant and susceptible grapevines were used, and these consisted of segregates that were a cross between *V*. *vinifera* cv. Airen with a hybrid of *V*. *rupestris* x. *V*. *arizonica* (b40-14 background). All plants were three-month-old grapevines and were rooted and established in one-gallon pots with a 5:1:1 mix of Supersoil: perlite: sand (Rod McLellan Company, San Mateo, CA, USA) and kept under controlled environmental conditions and watered to saturation at least once a week. Resistance was conferred via the *PdR1*c gene and verified by inoculation experiences as lacking disease symptoms and possessing low bacterial populations [9] to one of the genotypes used in this study, 07744–094. Another sibling (07744–092) was used in the study as a susceptible genotype for comparison, and it was from the same cross of the parents but was determined to lack the *PdR1c* gene. The 07744–092 displayed disease symptoms and high populations upon inoculation and was therefore considered susceptible.

### Phenolic compound level assessment in resistant and susceptible grapevines

Both the resistant and susceptible genotypes were either inoculated with *X*. *fastidiosa* spp. *fastidiosa*, mock-inoculated replacing the bacterial suspension with SCP buffer or left non-wounded as controls. Inoculations of *X*. *fastidiosa* were performed using the pin-prick method as based on the methods of Hill & Purcell [23]. Eight grapevines that were the susceptible genotype were inoculated with *X*. fastidiosa and eight grapevines that were the resistant genotype were inoculated with X. fastidiosa. For controls, eight grapevines that susceptible and eight grapevines that were resistant were mock inoculated with buffer or left non-wounded as controls. Four of each treatment combination were placed into one of two blocks to account for greenhouse micro-environment effects. A total of 48 plants total were utilized in these experiments (24 plants for each genotype).

At two-, five-, eight- and sixteen-weeks after the application of initial treatments, each grapevine had tissue samples collected. This involved taking two leaf blades from the base of the plant (for foliar samples) and collecting a 3 cm green stem segment (for woody tissue samples). Tissues then were immediately flash-frozen in liquid nitrogen. Samples were subsequently stored at -20°C until chemical analysis.

Metabolites were analyzed at the USDA-ARS in Parlier, CA according to procedures outlined in Wallis *et al*. [16, 24]. In brief, tissue samples were pulverized using a mortar and pestle and liquid nitrogen, with 0.10 g of tissue weighed into labeled 1.5 mL microcentrifuge tubes. Stem tissues were debarked prior to analyses to focus on the inner woody (xylem) tissues in which *X*. *fastidiosa* dwells. Pulverized, debarked wood tissue was analyzed chemically because previous work revealed that phenolic chemistry is similar in it to that present within the xylem sap that *X*. *fastidiosa* dwells [16–17]. Sample tubes had 0.5 mL of LC-MS grade methanol (from Fisher Scientific, Pittsburgh, USA) added and were incubated overnight at 4°C. The methanol was then removed to clean, labeled tubes, and another 0.5mL of methanol was added to the remaining pellet and incubated overnight at 4°C. The second extract was then removed and combined with the first to yield 1 mL of total methanol extract.

The methanol extracts subsequently were analyzed for phenolic compounds using a Shimadzu (Columbia, MD, USA) LC-20AD pump-based high-performance liquid chromatography equipped with a Supelco (Bellefonte, PA, USA) Ascentis C18 reverse phase column and a Shimadzu SPD-20 photodiode array detector. The running conditions were those in Wallis *et al*. [16], with a binary gradient running from 95% water (with 0.2% acetic acid) to 100% methanol (with 0.2% acetic acid) and back again over 40 min, and the column kept at 50°C. Compounds were putatively identified to at least compound subclass using a Shimadzu LCMS-2020 running the same gradient, matching UV/Vis maxima, and/or matching retention times with obtained standards from Sigma [16]. Compounds of the same type were converted to mg/g amounts using standard curves of ferulic acid (for hydroxycinnamic acid derivatives), quercetin glucoside (for flavonoids), catechin (for catechins and procyanidins), and piceid (for stilbenoids). A list of identification criteria for compounds is given in Table 1.

**Table 1:** Compounds quantified in this study with identification criteria provided.

Tissue	Final ID	Retention Time	Molecular Weight	UV/Vis Maxima	Compound Subclass
Leaf	caftaric acid derivative	8.67	312	294, 326	coumaric acid and tartaric acid derivative
Leaf	caftaric acid dimer	9.48	624	292, 327	coumaric acid and tartaric acid derivative
Leaf	chlorogenic acid derivative	10.58	354	279, 320	hydroxcinnamic acid
Leaf	caffeic acid glycoside	11.14	342	282, 316	hydroxcinnamic acid
Leaf	coutaric acid derivative 1	11.51	296	289, 311	coumaric acid and tartaric acid derivative
Leaf	coutaric acid dimer	12.54	592	290, 307	coumaric acid and tartaric acid derivative
Leaf	fetaric acid	13.64	326	276, 320	coumaric acid and tartaric acid derivative
Leaf	coutaric acid derivative 2	15.30	296	282, 310	coumaric acid and tartaric acid derivative
Leaf	uk^a^ flavonoid glycoside 1	15.72	464	278	flavonol glycoside
Leaf	uk flavonoid glycoside 2	16.83	448	279	flavonol glycoside
Leaf	*orientin*^b^	17.79	448	276	flavone glycoside
Leaf	uk flavonoid glycoside 3	18.37	480	270, 340	flavanonol glycoside
Leaf	uk flavonoid glycoside 4	18.90	494	265, 354	flavonol glycoside
Leaf	astragalin	19.22	448	280, 354	flavonol glycoside
Leaf	kaempferol-7-O-glucoside	19.43	448	279, 320	flavonol glycoside
Leaf	*rutin*	20.37	610	266, 354	flavonol glycoside
Leaf	*quercetin-3-O-glucoside*	20.67	464	255, 354	flavonol glycoside
Leaf	*quercetin glucuronide*	20.99	478	254, 356	flavonol glycoside
Leaf	*kaempferol-3-O-glucoside*	22.54	448	265, 346	flavonol glycoside
Leaf	kaempferol glucuronide	23.03	462	264, 348	flavonol glycoside
Wood	protocatechuic acid glycoside	8.20	316	284, 321	hydroxcinnamic acid
Wood	vanillic acid glycoside	9.10	330	284, 328	hydroxcinnamic acid
Wood	uk procyanidin dimer 1	9.89	578	278	procyanidin dimer
Wood	*procyanidin B1*	10.33	578	278	procyanidin dimer
Wood	procyanidin C2	10.60	866	278	procyanidin trimer
Wood	*catechin*	11.94	290	278	flavan-3-ol
Wood	*procyanidin B2*	12.49	578	278	procyanidin dimer
Wood	*procyanidin B2 gallate*	12.68	730	277	procyanidin dimer
Wood	*procyanidin C1*	13.23	866	277	procyanidin trimer
Wood	uk procyanidin dimer 2	14.17	578	278	procyanidin dimer
Wood	*epicatechin*	14.77	290	276	flavan-3-ol
Wood	epicatechin gallate	16.50	442	277	flavan-3-ol
Wood	uk procyanidin dimer gallate	16.98	730	277	flavonol glycoside
Wood	*piceid*	17.66	390	288, 316	stilbenoid glycoside
Wood	vitisin A	17.85	906	262, 320	resveratrol tetramer
Wood	miyabenol C	19.96	680	283, 354	resveratrol trimer
Wood	*quercetin-3-O-glucoside*	20.55	464	254, 266, 354	flavonol glycoside
Wood	*quercetin glucuronide*	21.09	478	306, 316	flavonol glycoside
Wood	vitisin B	21.77	906	306, 316	resveratrol tetramer
Wood	*episilon-viniferin*	23.78	454	282, 320	resveratrol dimer
Wood	piceatannol derivative	24.49	244	282, 325	hydroxystilbene

^a^uk = unknown.

^b^Compounds in italics were verified by comparison with commercial standards.

Additional pulverized leaf samples had DNA extracted by a DNAeasy plant kit (Macherey-Nagel, Bethlehem, PA, USA) according to Chen et al. [25], using the SDS version of the method and 0.10 g pulverized material. *X*. *fastidiosa* populations were determined by droplet digital PCR using mixes consisting of 5 μL of template DNA at a concentration of roughly 5 ng/μL, 1 μL each of primers at a concentration of 2 μM from Chen et al. [25], 12.5 μL of 2x ddPCR EvaGreen supermix (BioRad, Hercules, CA, USA), and 5.5 μL of ultrapure water per PCR reaction. Droplets were prepared by using the QX200 Droplet Generator (BioRad) with the reaction mix and EvaGreen Droplet Generator Oil (BioRad). PCR was conducted on the 40 μL droplet mix using a S1000 Thermocycler (BioRad) and an EvaGreen PCR method, including 40 total cycles with denaturing at 96°C for 30s, followed by annealing at 55°C for 30s, and extension at 72°C for 30s (based off Chen et al. [25]). Droplets then were read on a QX200 Droplet Reader (BioRad). The total number of positive droplets from the overall number of droplets per sample was determined using the Quantasoft software (BioRad). Standard curves were prepared for droplet digital PCR by extracting DNA from *X*. *fastidiosa* spp. *fastidiosa* stain Stags Leap grown in periwinkle wilt broth liquid culture, with an aliquot plated onto a periwinkle wilt solid agar plate to count the number of CFUs per mL. The dilution series of standard DNA allowed the conversion of droplet digital PCR counts (i.e. the number of positive droplets) to obtain estimates of bacterial CFUs per gram of leaf tissue, with calculated population values beneath 1 CFU/g tissue considered negative of *X*. *fastidiosa*.

All statistics were performed by SPSS version 24 (IBM, Armonk, NY, USA), and in all cases ɑ was set at 0.05. Due to normality assumptions not being met, especially the homogeneity of variances, comparisons of bacterial populations between resistant and susceptible grapevines were made using non-parametric Mann-Whitney U tests (used in cases where pairwise comparisons were made), and comparisons of bacterial populations between weeks were made with non-parametric Kruskal-Wallis tests (used in cases where comparisons were made between three or more groups). For both foliar and stem (woody tissue) phenolics, total phenolics were analyzed by summing together all individual compounds in the same sample. Likewise, compound subclasses (hydroxycinnamic acid derivatives, flavonoids, catechins and procyanidins, and stilbenoids) were assessed together by summing individual compounds in each of those classes. Analyses of variance were then performed on total phenolics or subclasses across all weeks (with the model having week collected, genotype, and infection treatment as independent variables with all interactions) and for each time individually (with genotype and infection treatment as independent variables with the interaction) to compare treatment effects. While samples collected from the same plant over time are not strictly independent, we chose not to model the repeated measures as a random effect because we had too few levels to adequately estimate the mean and variance of the random effect [26]. Following ANOVA, mean separations among treatments were made using least significant difference (LSD) tests.

To analyze compounds individually, multivariate analyses of variance (MANOVA) with Pillai’s trace Λ as the statistic were used with follow-up analyses of variance (ANOVA) and mean separations by least significant differences (LSD). This was done across all time points to assess the effect of time on the compounds, as well as for each collection time individually. Due to strong significant interactions of infection status and genotype with week (in most cases *P* < 0.001), separations for each time were noted and not overall across all weeks. A heat map was made to summarize the patterns of each compound across all treatments (week, genotype, and infection treatment) for each tissue. Due to substantial amount of information from the analyses of all individual compounds, details and statistics from these analyses are provided in S1 and S2 Tables.

### Fluctuations in *X*. *fastidiosa* populations over time and impacts of a second inoculation

To address the potential of *X*. *fastidiosa* population fluctuations influencing findings, separate, completely independent experiments were conducted to observe populations over time, as well as what occurs following a second infection that occurs after a previous infection. In June 2017, we needle inoculated a set of plants using two susceptible genotypes (07744–007 and 07744–092) and two resistant genotypes (07744–094 and 07744–102), for a total of four different genotypes. Inoculations of *X*. *fastidiosa* ssp. *fastidiosa* were performed using the pin-prick method as described by Hill and Purcell [23] into one point with 10 μL of a turbid suspension (greater than 1 OD_600_) of the STL isolate (American Type Culture Collection 700963) in SCP buffer. We repeatedly measured population size of live *X*. *fastidiosa* in petioles using serial dilution plating [23] at three, nine, and sixteen weeks post-inoculation. We then inoculated a random subset of these plants a second time at seventeen weeks post-inoculation, and inoculated an additional set of plants that were free of *X*. *fastidiosa*. This produced three treatments, with each of these having four grapevines from each of the four genotypes: 1) plants inoculated once in June; 2) plants inoculated once in October; and 3) plants inoculated twice. A total of 48 plants were used in the overall experiment. We then estimated *X*. *fastidiosa* population size from all plants at twenty-one and twenty-six weeks after the first inoculation date.

Statistics were performed in R 3.5.3 (R Core Team, Vienna, Austria). For the re-inoculation experiments, if resistant plants exhibited stronger induced resistance, we hypothesized that we would see the greatest decline in population size of *X*. *fastidiosa* in resistant plants that had been previously inoculated. As such, we focused our analysis on twenty-one and twenty-six-weeks post-inoculation and only for the two treatments inoculated in October. We tested for divergent slopes in *X*. *fastidiosa* population size between treatments and resistance trait. Because of low sample sizes, we combined genotypes based on the presence of resistance trait. We were specifically interested in the three-way interaction between week, treatment, and genotype. We used a generalized linear model with negative binomial error distribution because the *X*. *fastidiosa* population data were over-dispersed.

## Results

### Foliar tissue phenolic compound levels

Digital PCR confirmed that all non-inoculated and mock-inoculated plants were free of *X*. *fastidiosa*. For inoculated plants, bacterial populations were much lower in resistant grapevines (means and standard errors of 0.16±0.04 CFUs/g for week two, 0.02±0.01 CFUs/g for week five, 2.08±1.12 CFUs/g for week eight, and 0.12±0.04 CFUs/g for week sixteen) compared to susceptible grapevines (means and standard errors of 0.31±0.14 CFUs/g for week two, 41.64±21.85 CFUs/g for week five, 10.64±6.80 CFUs/g for week eight, and 40.14±16.31 CFUs/g for week sixteen). Mann-Whitney U tests confirmed resistant plants had significantly lower populations than susceptible plants (Mann-Whitney U = 108.00; *P* < 0.001). There was no effect of week on populations sizes (Kruskal-Wallis χ^2^ = 2.202; *P* = 0.532), that is, *X*. *fastidiosa* populations were not significantly different between weeks, likely due to interference from the effect of grapevine selection. Populations were variable, but for at least one timepoint all inoculated plants had values that met or exceeded 1 CFU/g. Symptoms were noted for susceptible grapevines and increased over time but were not present in resistant plants.

**Fig 1:**
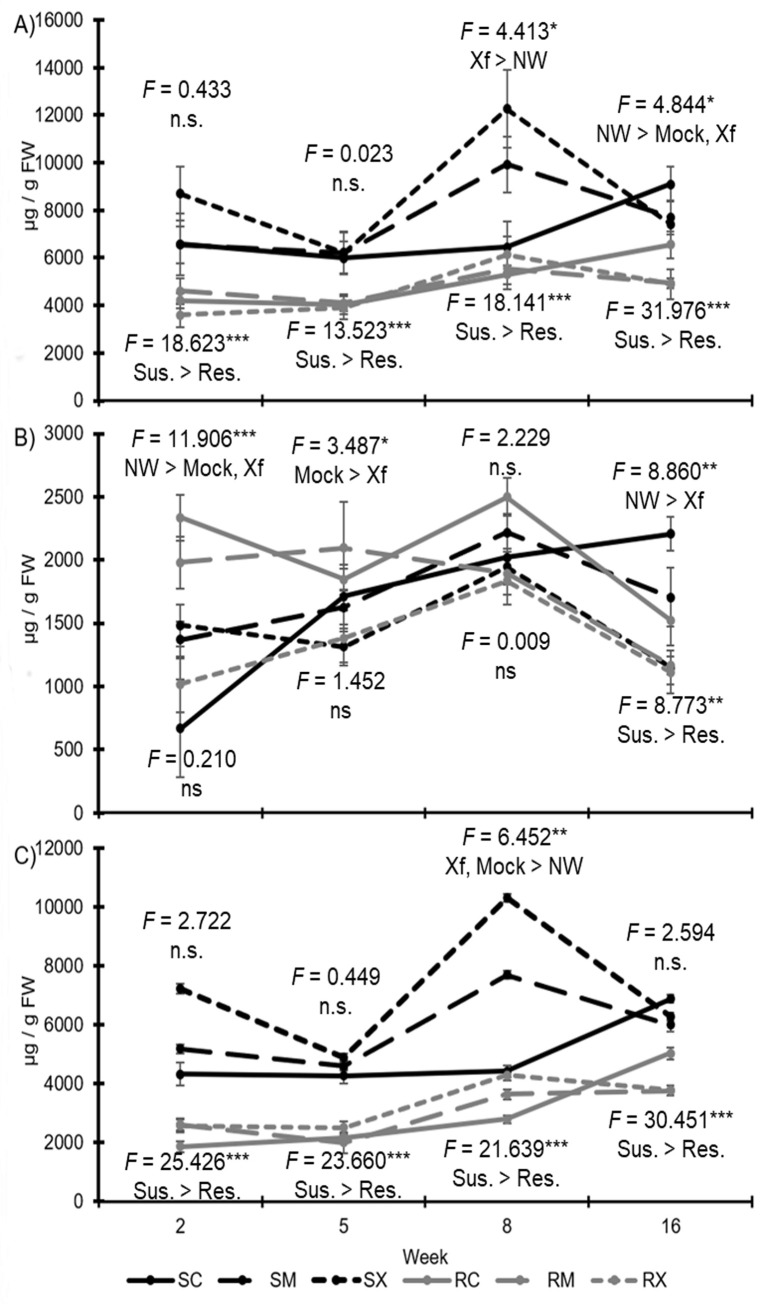
Phenolic levels in leaves of susceptible and resistant grapevines. Line graphs showing over time foliar levels of total phenolics (A), hydroxycinnamic acid derivatives (B), and flavonoids (C) for both resistant (R) and susceptible (S) grapevines receiving the different infection treatments [non-wounded (C), mock-inoculated (M), or *Xylella fastidiosa*-infected (X)]. F-statistics are provided for comparisons between infection treatments (top) and for genotypes (bottom), along with a brief description of significant effects by LSD. * represents p-values less than 0.050, ** represents p-values equal to or less than 0.010; *** represents p-values equal to or less than 0.001; n.s. = no significance; NW = non-wounded controls; Mock = mock-inoculated controls; Xf = *X*. *fastidiosa* inoculated plants; Sus. = susceptible genotype; Res. = resistant genotype.

The total of all phenolic compounds was significantly greater in susceptible than resistant sibling grapevines (*F*_1, 120_ = 72.107; *P* < 0.001; N = 144) (Fig 1A). Total phenolic levels peaked at eight-weeks after inoculation as levels were significantly greater than all other weeks (*F*_3, 120_ = 10.619; *P* < 0.001; N = 144). Phenolic levels at week sixteen were greater than week five as well. Total phenolics were not significantly different due to infection status. The ANOVA had a significant interaction of week and infection treatment (*F*_6, 120_ = 3.167; *P* = 0.006; N = 144), but no other significant interactions were observed.

When assessing all time periods individually, total phenolics were always greater in susceptible than resistant grapevines (for week two: *F*_1, 28_ = 18.623; *P* < 0.001; N = 34; for week five: *F*_1, 30_ = 13.523; *P* < 0.001; N = 36; for week eight: *F*_1, 31_ = 18.141; *P* < 0.001; N = 37; and for week sixteen: *F*_1, 31_ = 31.976; *P* < 0.001; N = 37) (Fig 1). In week eight infected grapevines had significantly greater total phenolic levels than non-wounded controls (*F*_2, 31_ = 4.413; *P* = 0.021; N = 37), whereas in week sixteen non-wounded controls had greater total phenolic levels than mock-inoculated or infected plants (*F*_2, 31_ = 4.844; *P* = 0.015; N = 37). There were no significant interactions when analyzing each week separately.

Foliar hydroxycinnamic acids (HCAs) were not significantly different in resistant compared to susceptible grapevines (*F*_1, 120_ = 0.102; *P* = 0.750; N = 144) (Fig 1B). However, HCAs were significantly affected by infection status (*F*_2, 120_ = 21.993; *P* < 0.001; N = 144), with greater HCA concentrations in non-infected controls and mock-inoculated plants than *Xylella*-infected plants. Likewise, week eight had greater HCA levels than all other weeks, and week two had greater HCA levels than week sixteen (*F*_3, 120_ = 9.795; *P* < 0.001; N = 144). There was a significant interaction between breeding line and week (*F*_3, 120_ = 3.039; *P* = 0.032; N = 144), and a significant three-way interaction between breeding line, time post-inoculation and infection status (*F*_6, 120_ = 2.639; *P* = 0.019; N = 144).

Examining each time period individually, in week sixteen susceptible grapevines had greater levels of HCAs than resistant ones (*F*_1, 31_ = 8.773; *P* = 0.006; N = 37), albeit for all other times levels were similar (Fig 1). For weeks two (*F*_2, 28_ = 11.906; *P* < 0.001; N = 34), eight (*F*_2, 31_ = 2.229; *P* = 0.125; N = 37), and sixteen (*F*_2, 31_ = 8.860; *P* = 0.001; N = 37) the non-wounded plants had significantly greater levels of HCAs than infected plants with multiple comparison tests (not significant by ANOVA for week eight), and for weeks two and five (*F*_2, 30_ = 3.487; *P* = 0.044; N = 36) the mock-inoculated plants had greater HCAs than infected plants. A significant treatment and genotype interaction was observed for week two only (*F*_2, 28_ = 3.857; *P* = 0.033; N = 34).

For foliar flavonoids, there were much greater levels in susceptible versus resistant grapevines (*F*_1, 120_ = 89.854; *P* < 0.001; N = 144) (Fig 1C). Likewise, levels in *X*. *fastidiosa* infected grapevines were greater than both the mock-inoculated and non-wounded controls (*F*_2, 120_ = 6.072; *P* = 0.003; N = 144). Flavonoid levels were significantly greater in weeks eight and sixteen than both weeks two and five (*F*_3, 120_ = 11.223; *P* < 0.001; N = 144). There was an interaction between infection status and genotype (*F*_2, 120_ = 3.171; *P* = 0.045; N = 144) and infection status and week (*F*_6, 120_ = 3.564; *P* = 0.003; N = 144).

For each week assessed individually, flavonoid levels were always greater in susceptible than resistant plants (for week two: *F*_1, 28_ = 25.426; *P* < 0.001; N = 34; for week five: *F*_1, 30_ = 23.660; *P* < 0.001; N = 36; for week eight: *F*_1, 31_ = 21.639; *P* < 0.001; N = 37; and for week sixteen: *F*_1, 31_ = 30.451; *P* < 0.001; N = 37) (Fig 1). Only for week eight did infected plants possess greater flavonoid levels than non-wounded controls (*F*_2, 31_ = 6.452; *P* = 0.005; N = 37), as the other weeks had similar levels among infection status. There were no significant interactions when examining flavonoids by separate weeks.

**Fig 2:**
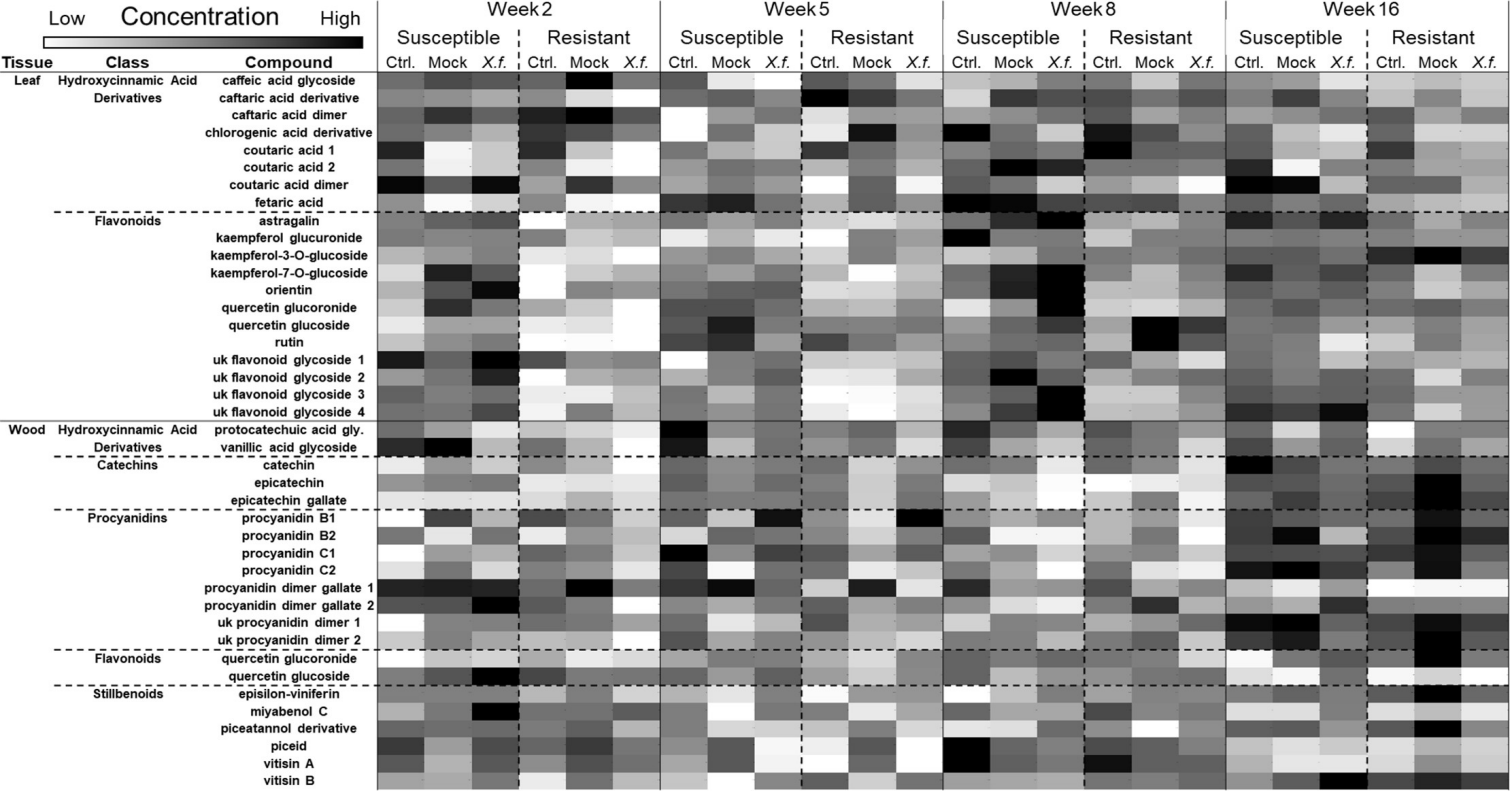
Visual representation of differences in individual phenolic levels of susceptible and resistant grapevines over time. Heat map showing patterns for levels of each individual compounds over time in both foliar and woody tissues, present in either resistant or susceptible grapevines, and in plants receiving the different infection treatments (non-wounded, mock-inoculated, or *Xylella fastidiosa*-infected), as a visible representation of the data provided in S1 and S2 Tables. Heat maps were done across all treatments and times, with lighter gray representing lowered levels, and darker gray representing increased levels. Refer to S1 and S2 Tables for greater details including standard errors of means and statistical test statistics.

Assessing individual compounds revealed that the majority of individual phenolics were present in greater amounts in the susceptible compared to resistant genotype (Fig 2; Table 2; S1 Table). Individual flavonoids especially exhibited this trend. The effect of infection status was more variable on phenolic levels, with some compounds increased in infected plants at certain times and reduced at other times (Fig 2; S1 Table). Every compound had levels very significantly affected by week (*P* < 0.001), with all but four compounds having the greatest levels in week eight and the least in week two (Fig 2; S1 Table).

**Table 2:** MANOVA statistics for analyses including all individual foliar or woody tissue compounds.

Tissue	Time Period	Main Effect/Interaction	Wilks Lambda	df1	df2	F	p
Foliar	All Weeks	week	2.138	3	120	12.769	<0.001
		genotype	0.680	1	120	10.749	<0.001
		infection status	0.996	2	120	5.005	<0.001
		week x genotype	1.209	3	120	3.477	<0.001
		week x infection	2.571	6	120	3.973	<0.001
		genotype x infection	0.575	2	120	2.056	<0.001
		week x genotype x infection	1.697	6	120	2.09	<0.001
	Week 2	genotype	0.849	1	28	2.529	0.077
		infection status	1.823	2	28	5.146	<0.001
		week x infection	1.656	2	28	2.407	0.019
	Week 5	genotype	0.840	1	30	2.877	0.038
		infection status	1.853	2	30	7.556	<0.001
		week x infection	1.774	2	30	4.711	<0.001
	Week 8	genotype	0.902	1	31	5.539	0.002
		infection status	1.867	2	31	9.144	<0.001
		week x infection	1.646	2	31	3.024	0.002
	Week 16	genotype	0.887	1	31	4.729	0.004
		infection status	1.506	2	31	1.98	0.035
		week x infection	1.348	2	31	1.344	0.215
Wood	All Weeks	week	1.803	3	131	6.666	<0.001
		genotype	0.400	1	133	2.888	<0.001
		infection status	0.717	2	132	2.446	<0.001
		week x genotype	0.814	3	131	1.649	0.003
		week x infection	1.623	6	128	1.695	<0.001
		genotype x infection	0.615	2	132	1.947	0.001
		week x genotype x infection	1.409	6	128	1.403	0.005
	Week 2	genotype	0.896	1	26	2.463	0.133
		infection status	1.507	2	26	1.02	0.511
		week x infection	1.692	2	26	1.828	0.111
	Week 5	genotype	0.940	1	27	5.248	0.016
		infection status	1.763	2	27	2.831	0.014
		week x infection	1.488	2	27	1.108	0.429
	Week 8	genotype	0.808	1	29	1.806	0.181
		infection status	1.586	2	29	1.822	0.075
		week x infection	1.534	2	29	1.566	0.141
	Week 16	genotype	0.815	1	29	1.888	0.163
		infection status	1.383	2	29	1.068	0.451
		week x infection	1.604	2	29	1.926	0.058

Separate MANOVAs were performed for all weeks together as well as individual weeks. Follow-up ANOVAs and means separations are provided for each compound in S1 and S2 Tables.

### Woody tissue phenolic compounds levels

**Fig 3:**
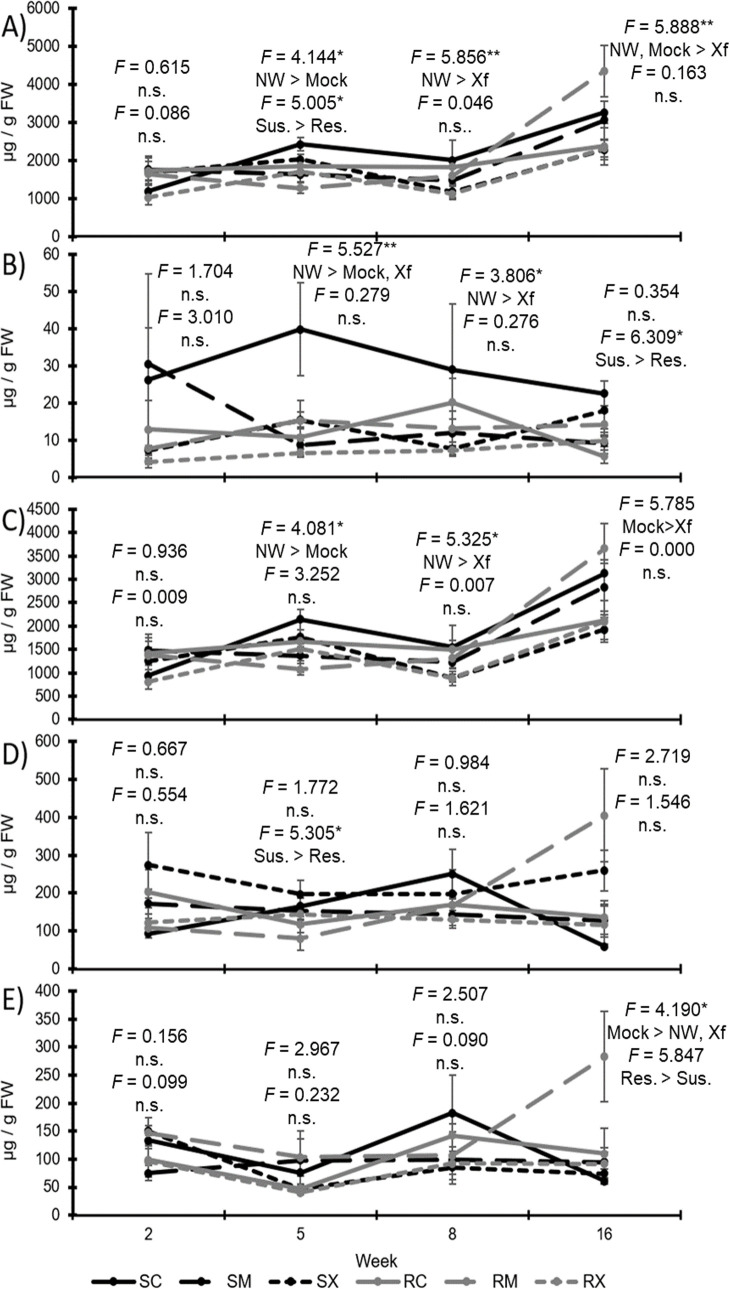
Phenolic levels in woody tissues of susceptible and resistant grapevines. Line graphs showing over time wood levels of total phenolics (A), hydroxycinnamic acid derivatives (B), flavonoids (C), catechins and procyanidins (D), and stilbenoids (E) for both resistant (R) and susceptible (S) grapevines receiving the different infection treatments [non-wounded (C), mock-inoculated (M), or *Xylella fastidiosa*-infected (X)]. F-statistics are provided for comparisons between infection treatments (top) and for genotypes (bottom), along with a brief description of significant effects by LSD. * represents p-values less than 0.050, ** represents p-values equal to or less than 0.010; *** represents p-values equal to or less than 0.001; n.s. = no significance; NW = non-wounded controls; Mock = mock-inoculated controls; Xf = *X*. *fastidiosa* inoculated plants; Sus. = susceptible genotype; Res. = resistant genotype.

Total phenolic levels from woody tissues between resistant and susceptible plants did not clearly differ (*F*_1, 111_ = 0.545 *P* = 0.462; N = 135) (Fig 3A). However, levels were greater in non-wounded and mock-inoculated controls than in *X*. *fastidiosa*-infected plants (*F*_2, 111_ = 5.287; *P* = 0.006; N = 135). Total phenolic levels also were greater at sixteen weeks than at all other weeks (*F*_3, 111_ = 27.327; *P* < 0.001; N = 135). The only significant interaction was between infection status and week (*F*_6, 111_ = 3.884; *P* = 0.001; N = 135).

Analyzing weeks separately, it was observed that susceptible plants possessed greater wood total phenolic levels for week five only (*F*_1, 27_ = 5.005; *P* = 0.034; N = 33) (Fig 3). Non-wounded plants were similar to infected plants in week two but possessed greater total phenolic levels in weeks five (*F*_2, 27_ = 4.144; *P* = 0.027; N = 33), eight (*F*_2, 29_ = 5.856; *P* = 0.007; N = 35), and sixteen (*F*_2, 29_ = 5.888; *P* = 0.007; N = 35). Mock-inoculated plants had greater total phenolic levels than infected plants in week sixteen as well. There were no observed significant interactions (*P* > 0.05).

Woody tissue hydroxycinnamic acid (HCA) derivatives occurred at greater levels in susceptible compared to resistant plants (*F*_1, 111_ = 10.917; *P* = 0.001; N = 135) and were greater in non-wounded plants than those mock-inoculated or infected (*F*_2, 111_ = 6.936; *P* = 0.001; N = 135) (Fig 3B). Week collected did not affect HCA levels. There were no significant interactions in the analyses.

Examining individual collection times, at only weeks five (*F*_1, 27_ = 7.632; *P* = 0.010; N = 33) and sixteen (*F*_1, 29_ = 6.309; *P* = 0.018; N = 35) did susceptible plants have significantly greater HCA levels than resistant plants (Fig 3). For weeks five (*F*_2, 27_ = 5.527; *P* = 0.010; N = 33) and eight (*F*_2, 29_ = 3.806; *P* = 0.034; N = 35) non-wounded plants possessed greater HCA levels than infected plants. There were significant interactions between infection status and genotype for weeks five (*F*_2, 27_ = 6.636; *P* = 0.005; N = 33) and sixteen (*F*_2, 29_ = 5.447; *P* = 0.010; N = 35).

For wood flavonoids (those that were not flava-3-ols or procyanidins), there were no significant effects due to genotype, infection status, or week (Fig 3C). Significant interactions were observed for genotype by infection status (*F*_2, 111_ = 4.959; *P* = 0.009; N = 135) and genotype by infection status by week (*F*_6, 111_ = 2.686; *P* = 0.018; N = 135). For analyses of each week separately, the only significant differences observed were greater levels in susceptible versus resistant plants in week five (*F*_1, 27_ = 5.305; *P* = 0.029; N = 33), and a significant genotype by infection status interaction in week sixteen (*F*_2, 29_ = 5.276; *P* = 0.011; N = 35). It should be noted that far fewer non-procyanidin associated flavonoids were observed in wood tissues than leaf tissues.

For wood catechins (flava-3-ols) and derivative procyanidins, there were greater levels in both non-wounded and mock-inoculated plants than those that were *X*. *fastidiosa*-infected (*F*_2, 111_ = 5.831; *P* = 0.004; N = 135) (Fig 3D). Level were greater at week sixteen than all other weeks, and greater in week five than weeks two or eight (*F*_3, 111_ = 32.070; *P* < 0.001; N = 135). There were no significant differences between susceptible and resistant grapevines (*F*_1, 111_ = 0.544; *P* = 0.462; N = 135). There was a significant interaction between infection status and week (*F*_6, 111_ = 3.505; *P* = 0.003; N = 135). Analyses of each week separately observed differences due to infection status for weeks five (*F*_2, 27_ = 4.081; *P* = 0.028; N = 33), eight (*F*_2, 29_ = 5.325; *P* = 0.011; N = 35), and sixteen (*F*_2, 29_ = 5.785; *P* = 0.008; N = 35), with week five having greater levels in non-wounded controls versus mock-inoculated plants, week eight having greater levels in non-wounded plants than those that were infected, and week sixteen having greater levels in mock-inoculated plants than infected plants. There were no observed differences due to genotype or significant interactions.

For wood stilbenoids, greater levels were present in mock-inoculated than *Xylella*-infected plants (*F*_2, 111_ = 3.579; *P* = 0.031; N = 135) (Fig 3E). Levels in week five were significantly lower than all other weeks (*F*_3, 111_ = 3.386; *P* = 0.021; N = 135). There were no differences between susceptible and resistant plants (*F*_1, 111_ = 1.300; *P* = 0.257; N = 135). Significant interactions were observed between genotype and infection status (*F*_2, 111_ = 3.963; *P* = 0.022; N = 135), genotype and week (*F*_3, 111_ = 3.092; *P* = 0.030; N = 135), and infection status and week (*F*_6, 111_ = 2.750; *P* = 0.016; N = 135). When analyzing each week separately, greater stilbenoid levels were present in resistant versus susceptible plants for week sixteen only (*F*_1, 29_ = 5.847; *P* = 0.022; N = 35). Mock-inoculated plants had greater levels than non-wounded or infected plants in week sixteen as well (*F*_2, 29_ = 4.190; *P* = 0.025; N = 35). For other weeks, treatments were not statistically different. The only significant infection status by genotype interaction was observed in week two (*F*_2, 26_ = 4.558; *P* = 0.002; N = 32).

Assessing individual compounds revealed that the majority of individual phenolics were not usually present in greater amounts in the susceptible compared to resistant genotype except in week five (Fig 2; Table 2; S2 Table). Likewise, week five was the only time infection status affected compound levels, with varying effects dependent on the compound (S2 Table). More individual compounds had greater levels in week sixteen than earlier weeks (S2 Table).

### Re-inoculation experiments

**Fig 4:**
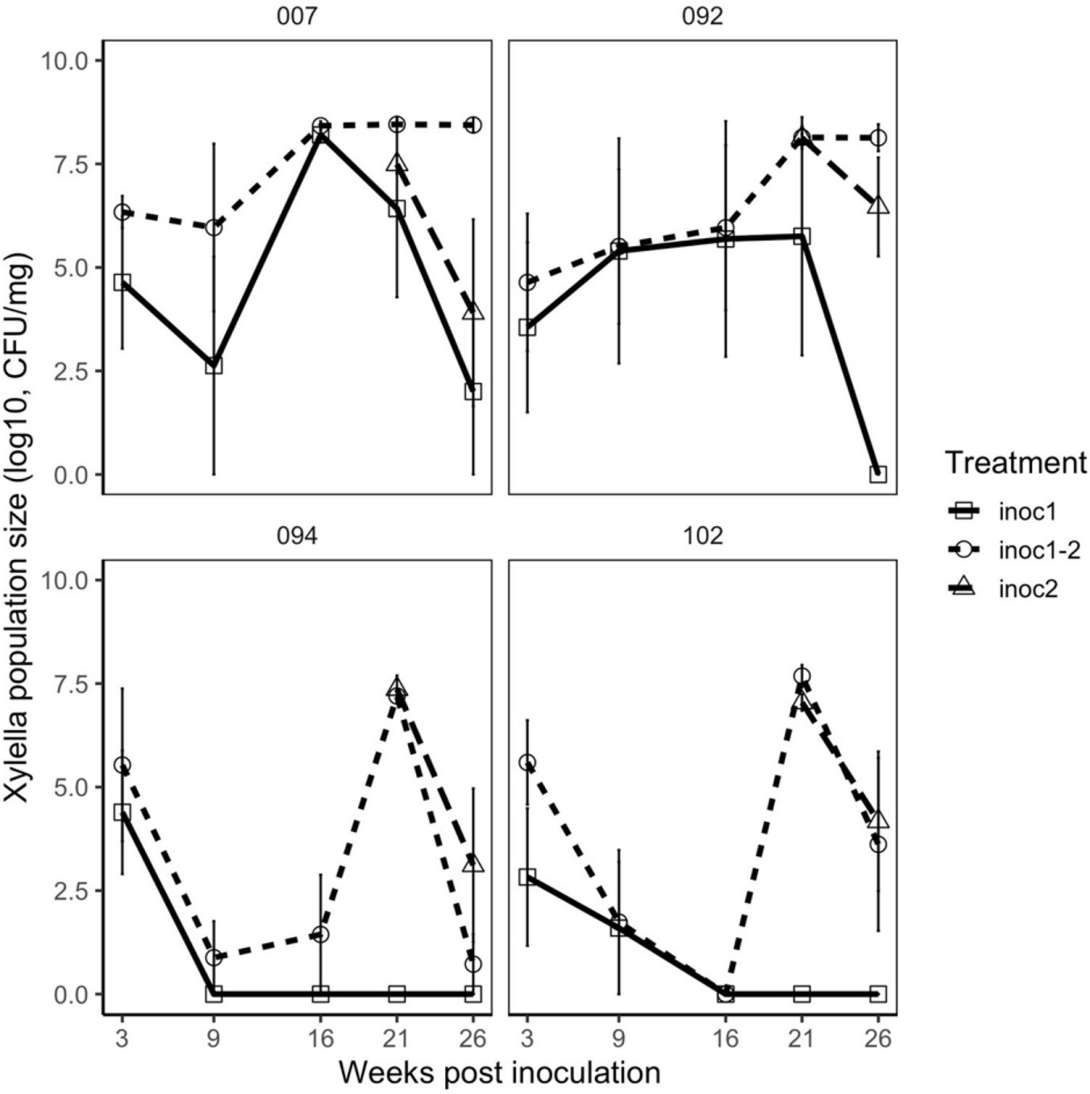
*Xylella fastidiosa* population sizes in the reinfection experiment over time. Complete timeseries for *X*. *fastidiosa* population size estimates from the reinfection experiment, measured over weeks since the first inoculation. Treatments include “inoc1” where plants were inoculated only at the first inoculation date; “inoc2” where plants were inoculated only at the second inoculation date; and “inoc1-2” where plants were inoculated at both inoculation dates. Second inoculation occurred seventeen weeks after the first inoculation. Panels represent the different genotypes used in the experiment with Susceptible genotypes on the top row (007 and 092) and Resistant genotypes on the bottom row (094 and 102).

We tested for differences in population size of *X*. *fastidiosa* after either one or two inoculation events, spaced seventeen weeks apart using all four genotypes. We found a clear overall reduction in population size between twenty-one weeks and twenty-six weeks from the first inoculation (coefficient estimate [95% CI] = -0.467 [-0.922, -0.0121], *t* = -2.20, *P* = 0.028) (Fig 4). We were particularly interested in the three-way interaction between week, treatment, and resistance trait. In line with our hypothesis, resistant genotypes that had been inoculated twice showed the greatest decline in *X*. *fastidiosa* population size (Fig 4). However, this trend could not be detected statistically (coefficient estimate [95% CI] = -0.306 [-1.16, 0.547], *t* = -0.719, *P* = 0.472).

## Discussion

Often sources of resistance are discovered and deployed in breeding programs with no understanding about the mechanism of how said resistance genes or loci function. Such knowledge could help better understand plant-pathogen interactions as well as allow improved understanding about the durability of resistance, with genes controlling physiological mechanisms to restrict pathogens or ameliorate disease likely far more durable than specific gene-for-gene responses.

Towards this end, observations of an important class of plant defense and repair compounds, phenolics, were made over the course of *X*. *fastidiosa* infection in the woody and foliar tissues of grapevines. Although few differences were observed between resistant grapevines containing the *PdR1* locus and susceptible siblings lacking it in the wood, concentrations of total phenolics in leaves of resistant plants were about half of those found in leaves of susceptible plants. In particular, flavonoid levels were far lower in leaves from resistant plants than susceptible plants. Levels were most different in the foliage likely due to leaves being far more flexible and productive overall than woody tissues, which have far fewer cells capable of making phenolics as most are xylem elements or pith cells.

That flavonoid levels were at far lower levels in *PdR1* resistant plants was surprising, as these compounds are often associated with antibiotic activity against certain microbes [27]. Flavonoids have been described as having activities to reduce bacterial populations by disrupting cell membranes, inhibiting nucleic acid synthesis, inhibiting ATP synthesis, disrupting mineral acquisition by acting as chelators, and inhibition of bacterial toxin production [27]. Yet, these results and those previously reported in Wallis & Chen [16] and Wallis *et al*. [17] observed phenolic levels, likely flavonoid levels, were consistently reduced in grapevine cultivars exhibiting lowered susceptibility to *X*. *fastidiosa*.

Reduced flavonoid concentrations present in xylem tissues that *X*. *fastidiosa* encounters early in infections could be important in establishment of the bacteria within its host and key for later development of disease. *X*. *fastidiosa* has a “two-form” infection process, whereby the bacteria either exist as a free-flowing planktonic lifestyle to rapidly colonize plant tissues far from the inoculation site, or exist with an aggregated, biofilm lifestyle that leads to symptom formation [28]. This, in turn, results in far more success in causing Pierce’s disease as a greater proportion of the host is colonized prior to the aggregation phase. Indeed, previous studies have observed the effect of phenolic compounds reducing bacteria biofilm formation [27, 29], albeit not always [30]. Further, flavonoids may reduce biofilms by reducing quorum sensing [29, 31], likely the perception of the diffusible signal factor, and the mechanism may be related to the chelating properties of these compounds [32]. These results therefore lead to a hypothesis that innate flavonoids present in susceptible grapevines may cause a greater proportion of *X*. *fastidiosa* to adopt the planktonic lifestyle in grapevine hosts upon infection, allowing more thorough, systemic colonization of the grapevine. Culture-based studies examining the affects of flavonoids on *X*. *fastidiosa* are thereby warranted to provide further support to this hypothesis.

This study further observed what occurs if a previously infected grapevine becomes re-inoculated later in the growing season. Our results suggest that resistant grapevines had a predictable and more rapid reduction of *X*. *fastidiosa* populations when re-infected. However, the trend was not statistically significant. This conclusion could have been influenced by late season phenological changes in phenolic compound levels. Although phenolics were not assessed past week 16 due to an emphasis on one infection at a time, by week sixteen a clear trend was observed whereby cell wall compounds such as hydroxycinnamic acid derivatives (HCAs) appeared to increase in all grapevines studied, regardless of genotype or infection status. This was likely due to phenological changes in grapevine previously observed by Wallis & Chen [16] whereby tissue thickening occurs prior to senescence. Thicker cell walls could have limited *X*. *fastidiosa* spread within its host and its populations accordingly in all grapevines of this experiment. Stilbenoids levels were greater in the resistant genotype in week sixteen than the susceptible genotype, and this could explain a more rapid decline in observed secondary infection *X*. *fastidiosa* populations, even if it was not a significantly different one.

Overall, these results observed differences in a compound class important in host defense, phenolics, between resistant and susceptible grapevine siblings. We hypothesize that the presence of the *PdR1* locus, originally from a Pierce’s disease resistant wild species of grapevine *V*. *arizonica*, results in reduced flavonoid levels. Furthermore, we hypothesized that such reductions in flavonoids may have affected *X*. *fastidiosa* infections by altering the bacterial lifestyle, albeit additional studies are warranted. Such a trait could be assessed in other potential resistance-imparting loci to determine whether phenolic alterations are a resistance mechanism to this and similar pathogens.

## Supporting information

S1 Table Analyses of individual compounds in leaves of susceptible and resistant grapevines over time. Leaf tissue compound mean (± SE) levels (μg / g FW) for each treatment combination (phenotype by inoculation treatment) per each week individually. ANOVA statistics are provided, as well as a description of significant effects or LSD separations. HCAs = hydroxycinnamic acid derivatives; FLAVs = flavonoids; Res = resistant genotype; Sus = susceptible genotype; None = non-infected controls; Mock = mock-inoculated plants; Xf = X. fastidiosa infected plants.
(XLSX)

S2 Table Analyses of individual compounds in woody tissues of susceptible and resistant grapevines over time. Woody tissue compound mean (± SE) levels (μg / g FW) for each treatment combination (phenotype by inoculation treatment) per each week individually. ANOVA statistics are provided, as well as a description of significant effects or LSD separations. HCAs = hydroxycinnamic acid derivatives; CATs = catechins; PROs = procyanidins; FLAVs = flavonoids; STILLs = stilbenoids; Res = resistant genotype; Sus = susceptible genotype; None = non-infected controls; Mock = mock-inoculated plants; Xf = X. fastidiosa infected plants.
(XLSX)
